# From Inflammation to Dysfunction: The Impact of a First Acute Pancreatitis Episode on Pancreatic Function

**DOI:** 10.3390/jcm14144932

**Published:** 2025-07-11

**Authors:** Marina Balaban, Daniel Vasile Balaban, Iulia Enache, Ioan Cristian Nedelcu, Mariana Jinga, Cristian Gheorghe

**Affiliations:** 1 Doctoral School, Carol Davila University of Medicine and Pharmacy, 020021 Bucharest, Romania; marina.ciochina@drd.umfcd.ro (M.B.); iulia.enache@drd.umfcd.ro (I.E.); ioan-cristian.nedelcu@drd.umfcd.ro (I.C.N.); 2 Internal Medicine and Gastroenterology Department, Carol Davila University of Medicine and Pharmacy, 020021 Bucharest, Romania; mariana.jinga@umfcd.ro (M.J.); cristian.gheorghe@umfcd.ro (C.G.); 3 Gastroenterology Department, Central Military Emergency University Hospital, 010825 Bucharest, Romania; 4 Gastroenterology Department, Fundeni Clinical Institute, 022328 Bucharest, Romania

**Keywords:** acute pancreatitis, pancreatic exocrine insufficiency, pancreatic endocrine insufficiency, diabetes mellitus

## Abstract

**Background/Objectives**: The complexity of acute pancreatitis (AP) extends beyond its immediate complications. This study aimed to evaluate both exocrine and endocrine pancreatic dysfunctions following a first episode of AP, assessed at diagnosis and during a 6-month follow-up period. **Methods**: A prospective analysis was conducted on patients with a first episode of AP. Pancreatic endocrine function was evaluated using fasting glucose and glycated hemoglobin (HbA1c) levels, while pancreatic exocrine function was assessed through fecal elastase-1 (FE-1) testing and the novel Pancreatic Exocrine Insufficiency Questionnaire (PEI-Q). **Results**: Altogether, data from 112 time-point observations were analyzed with respect to endocrine and exocrine insufficiency after a first episode of AP, with 60 patients enrolled at baseline, 33 (55%) completing the first follow-up, and 19 (31.67%) completing the second follow-up. Based on PEI-Q scores, 75% of patients showed pancreatic exocrine insufficiency (PEI) at baseline. This rate decreased significantly to 33.3% at 2 months, with a further slight decline to 26.3% at 6 months. In contrast, FE-1 testing identified PEI in only 23% of patients at baseline, with a similar progressive improvement in time. Regarding the endocrine function, hyperglycemia was noted at baseline (mean serum glucose 120.75 ± 49.89 mg/dL), with a decreasing trend and normalization observed at follow-up. **Conclusions**: The pancreas has a remarkable recovery potential, with both exocrine and endocrine dysfunctions seen during the hospitalization for AP being transient. However, follow-up after AP is essential, as pancreatic insufficiency can significantly impact patients’ quality of life.

## 1. Introduction

The complexity of acute pancreatitis (AP) extends beyond its acute phase. Growing attention is being devoted to its late complications and to the importance of long-term follow-up. These late complications involve, on one hand, impairment of the pancreas’s exocrine function—critical for digestion through pancreatic enzyme secretion—and, on the other hand, dysfunction of its endocrine function, increasing the risk of glycemic dysregulations, prediabetes, and diabetes mellitus (DM).

The close relationship between the exocrine and endocrine pancreas starts within organogenesis and persists throughout life, ensuring both normal pancreatic function and contributing to the pathogenesis of certain diseases [1,2,3].

AP is the leading pancreatic condition associated with the onset of type 3c diabetes mellitus (T3cDM) [4,5]—a secondary form of diabetes caused by exocrine pancreatic dysfunction—through mechanisms such as islet cell destruction due to inflammation and necrosis, altered insulin signaling [6], Il-6-mediated insulin resistance [7], and enhanced lipolysis [7], with longitudinal data showing that up to 40% of patients develop diabetes within five years after a first AP episode [8].

Pancreatic exocrine insufficiency (PEI) following AP results from both structural damage—such as acinar cell loss, ductal obstruction, and fibrosis—and functional dysregulation of hormonal and neural control of enzyme secretion [9], with meta-analyses reporting a prevalence ranging from 27% to 62%, and long-term persistence in approximately one-third of cases [9,10]. Fecal elastase-1 (FE-1), while widely used for diagnosing PEI, has known limitations, including reduced sensitivity in mild-to-moderate disease and potential false positives due to watery stools [11].

In the present study, we aimed to assess the presence of both pancreatic exocrine and endocrine insufficiency following a first episode of AP. We further investigated the correlation between specific biological markers, such as FE-1, and the novel PEI-Q score in order to evaluate their potential utility in the early diagnosis of PEI.

## 2. Materials and Methods

### 2.1. Study Design

We enrolled patients admitted with a diagnosis of AP between 1 May 2023 and 1 December 2024. The diagnosis of AP was established in accordance with current international diagnostic recommendations, requiring the presence of at least two of the following three criteria: (1) characteristic abdominal pain; (2) serum lipase or pancreatic amylase levels greater than three times the upper limit of normal; and (3) imaging findings consistent with AP on abdominal ultrasonography, computed tomography (CT), or magnetic resonance imaging (MRI) [12]. AP severity was defined according to the Revised Atlanta Classification.

### 2.2. Participants

Eligible participants were adults (≥18 years) diagnosed with a first episode of AP and without a prior diagnosis of PEI, willing to complete the consent form. Exclusion criteria included: known pre-existing PEI (documented by previous FE-1 measurement or other diagnostic methods), prior diagnosis of chronic pancreatitis (CP) (confirmed by imaging: abdominal ultrasound, computed tomography—CT/magnetic resonance imaging—MRI, or endoscopic ultrasonography—EUS), diagnosis of pancreatic malignancy (confirmed by imaging or histopathological analysis), or history of previous AP.

### 2.3. Work-Up of Enrolled Patients

At the time of initial hospitalization for AP, the following baseline biological parameters were recorded: leukocyte count, the peak value of C-reactive protein (CRP) during hospitalization, fasting blood glucose and glycated hemoglobin (HbA1c), and FE-1. Radiological severity was assessed using the Balthazar score and the Computed Tomography Severity Index (CTSI). Clinically, symptoms suggestive of PEI were evaluated using the PEI-Q score.

The PEI-Q score is a questionnaire-based tool, a patient-reported outcome (PRO) developed to quantify symptoms associated with PEI and their effect on quality of life. It includes 18 items grouped in 3 domains—(A) abdominal symptoms, (B) bowel movement symptoms, and (C) impact on quality of life. The PEI-Q score is obtained by calculating the total symptom score, assessed by calculating the mean score of symptoms in domains A and B. Based on PEI-Q scores, patients were stratified into four groups: <0.6 (no PEI), 0.6–1.39 (mild PEI), 1.4–1.79 (moderate PEI), and ≥1.8 (severe PEI). Only patients with a PEI-Q score greater than or equal to 0.6 can complete the impact domain, and only for them can the total summary score be calculated. The score has been validated in assessing PEI in several cohorts [13,14,15]. The Romanian translation of the PEI-Q score, which we used in the current research, is available in the Supplementary Materials.

**Figure 1:**
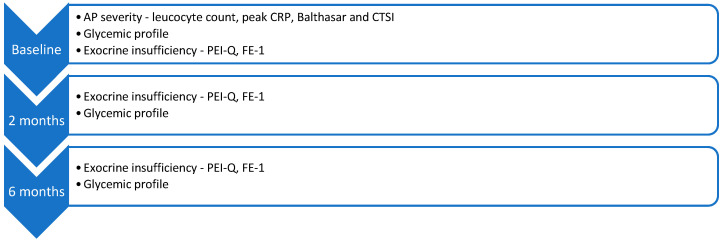
Algorithm for patient assessment at AP diagnosis and follow-up.

At the first follow-up, conducted at two months after the initial AP episode, the following serological variables were reassessed: fasting glucose, HbA1c, CRP, and serum albumin. The same parameters were evaluated at the second follow-up, approximately six months post-AP. Also, PEI-Q and FE-1 were reassessed at both follow-up time points (Figure 1).

FE-1, as a marker of PEI, was measured at baseline and during both follow-up visits. Based on FE-1 levels, patients were categorized into three groups: severe insufficiency (<100 µg/g), moderate insufficiency (100–199 µg/g), and normal exocrine function (≥200 µg/g) [16].

Elevated leukocyte counts were defined as values exceeding the hospital laboratory’s upper reference limit (>11 × 10^3^/µL). Hyperglycemia was defined as fasting glucose levels ≥100 mg/dL. According to the HbA1c level, patients were classified into three categories based on the American Diabetes Association guidelines: normoglycemic (HbA1c < 5.7%), prediabetic (HbA1c 5.7–6.4%), and diabetic (HbA1c ≥ 6.5%) [17]. Elevated CRP was considered in values exceeding the hospital laboratory’s upper reference limit (>5 mg/L), while low albumin level was considered <3.5 g/dL.

### 2.4. Statistical Analysis

Statistical analyses were performed using IBM SPSS Statistics version 29, and graphical representations were created with Microsoft Excel and Microsoft Word version 16.77. Quantitative variables were presented as means with standard deviations. Categorical variables were reported as absolute values and percentages. Missing outcome data regarding FE-1 levels at baseline, 2, and 6 months were addressed using multiple imputation and the expectation maximization method (EM), an algorithm that finds maximum likelihood estimates of parameters in statistical models. A total of 40 missing values were imputed across all time points. In order to compare the FE-1 levels and PEI-Q scores (baseline, 2 months, 6 months), a general linear model for repeated measures was used. A univariate analysis for repeated measures ANOVA analyzed the variance of PEI-Q and FE-1 among the three moments. A *p*-value of <0.05 was considered statistically significant for all analyses. Internal consistency of the PEI-Q questionnaire was assessed at baseline, 2 months, and 6 months using Cronbach’s α. Values above 0.70 were considered indicative of acceptable internal reliability.

### 2.5. Ethics

Participation in the study was voluntary and preceded by the signing of written informed consent. Ethical approval was obtained from the Ethics Committee of the Central Military Emergency University Hospital “Dr. Carol Davila,” Bucharest (approval no. 489/28.01.2022).

## 3. Results

**Figure 2:**
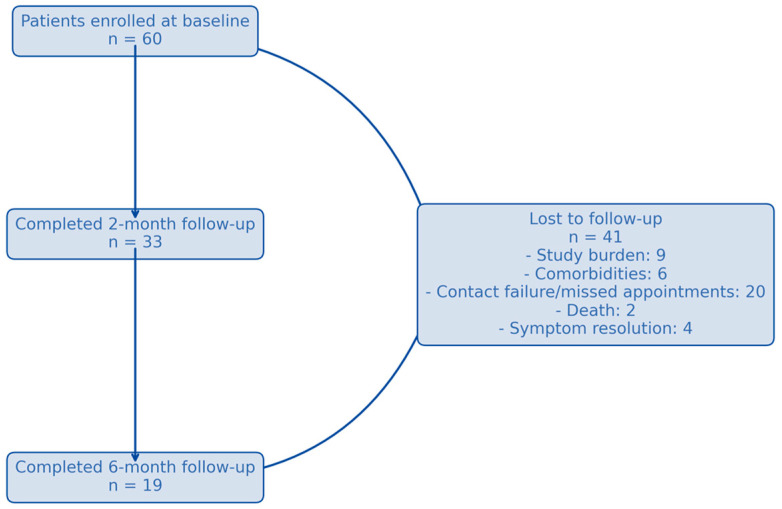
Patient flow diagram.

Altogether, the dataset included 112 time-point observations, which were analyzed with respect to endocrine and exocrine insufficiency after a first episode of AP, with 60 patients enrolled at baseline, 33 (55%) completing the first follow-up, and 19 (31.67%) completing the second follow-up (Figure 2).

### 3.1. At Presentation

A total of 60 patients were recruited at presentation, with a mean age of 57.18 ± 13.3 years. The gender distribution was balanced (48.3% males). Regarding risk factors, 25% of patients were smokers and 35% reported alcohol consumption. General demographics, laboratory parameters, and imaging characteristics at the time of first hospitalization are summarized in Table 1.

Among the 60 included patients at baseline, most were biliary or alcoholic AP, followed by hypertriglyceridemia-associated AP and post-ERCP pancreatitis, and there was one case of IgG4-related AP.

Fecal elastase-1 (FE-1) was measured at AP onset in 26 patients. Among them, the majority had values exceeding 200 µg/g, indicating preserved exocrine function. Due to the impossibility of early stool sampling after AP onset and potential confounders, including AP-related therapy, FE-1 was not assessed in the remaining patients.

In questionnaire assessment, based on PEI-Q questionnaire scores at baseline, 15 patients (25%) had no evidence of PEI, while 30 patients (50%) were classified as having mild PEI, 9 patients (15%) moderate PEI, and 6 patients (10%) severe PEI.

**Figure 3:**
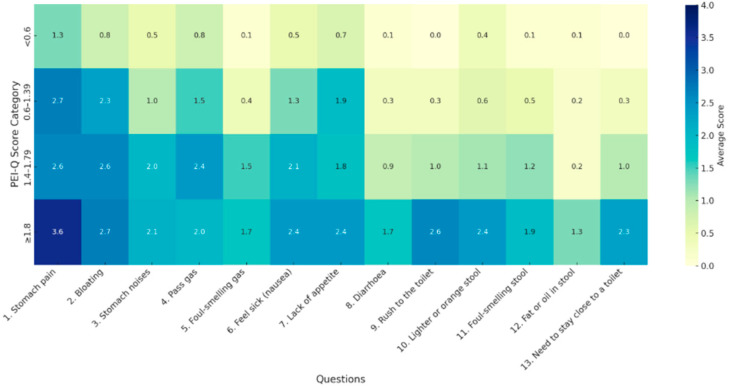
Heatmap of mean responses to PEI-Q questionnaire at presentation.

Analysis of responses to the PEI-Q questionnaire revealed that patients with scores ≥1.8 more frequently reported the following symptoms: abdominal pain, bloating, fecal urgency, changes in stool color, nausea, and lack of appetite (Figure 3).

**Figure 4:**
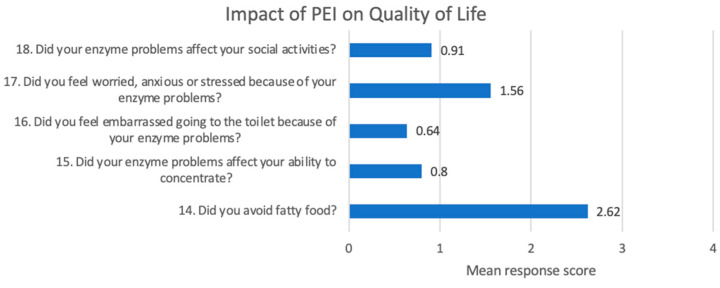
Impact of PEI on quality of life—first hospitalization.

Patients with PEI as defined by a PEI-Q score ≥ 0.6 also completed the items assessing the impact of PEI on quality of life. The burden of PEI was evaluated across five key dimensions. The most affected domain was avoidance of fatty food consumption, with a mean score of 2.62, indicating a significant adaptive behavioral response to gastrointestinal symptoms. In addition, worry, distress, or stress related to enzyme-related digestive problems had a mean score of 1.56, suggesting a notable impact on psychological well-being. Other aspects—such as concentration difficulties (mean score: 0.80), feelings of embarrassment (0.64), and limitations in social activities (0.91)—were rated lower, indicating a more moderate impact on cognitive and social functioning (Figure 4).

At baseline, PEI-Q is negatively correlated with FE-1, without statistical significance (*p* = 0.678), suggesting that as the severity of exocrine pancreatic insufficiency increases, reflected by higher PEI-Q scores, FE-1 levels tend to decrease. Notably, patients classified as having severe PEI (PEI-Q score ≥ 1.8) demonstrated markedly lower FE-1 values compared to those with mild or moderate PEI.

Patients with FE-1 values below 200 µg/g most commonly reported abdominal pain as the predominant symptom on the PEI-Q questionnaire, followed by nausea, flatulence, and bloating. In contrast, those with FE-1 levels below 100 µg/g primarily reported abdominal pain, nausea, loss of appetite, and bloating.

### 3.2. First Follow-Up Assessment (2 Months Post AP)

Of the patients included in the study, 33 (55%) attended the two-month follow-up visit. At this time point, 27.3% of patients demonstrated elevated fasting blood glucose levels, 40% had elevated CRP, and 15.2% had FE-1 values below 200 μg/g (Table 2). Follow-up cross-sectional imaging was performed in 20 patients, with the majority classified as Balthazar score A (45%).

Regarding the PEI-Q questionnaire, the mean PEI-Q score was 0.6 ± 0.52, and the majority (66.7%) reported scores below 0.6.

**Figure 5:**
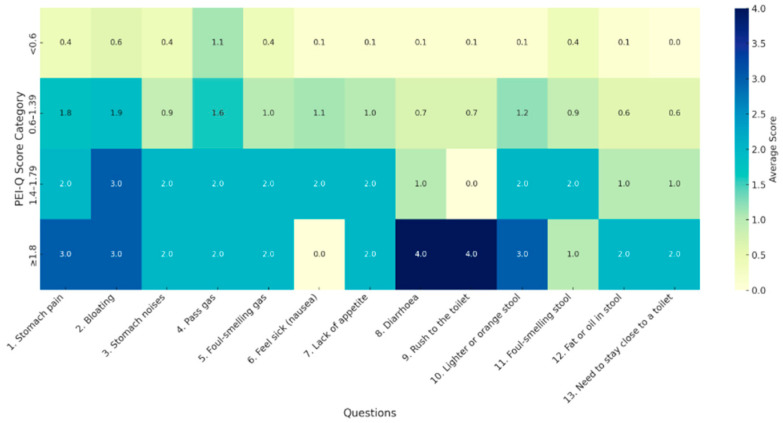
Heatmap of mean responses to PEI-Q questionnaire at 2-month follow-up.

At this time point, only 15% presented decreased FE-1. The PEI-Q questionnaire identified 66.7% of patients without PEI, 27.3% with mild PEI, 3% with moderate PEI, and 3% with severe PEI. We further analyzed the mean responses from the PEI-Q questionnaire, stratifying them according to PEI-Q score categories. A progressive increase in symptom severity was observed across PEI categories, particularly among patients with severe PEI (Figure 5).

**Figure 6:**
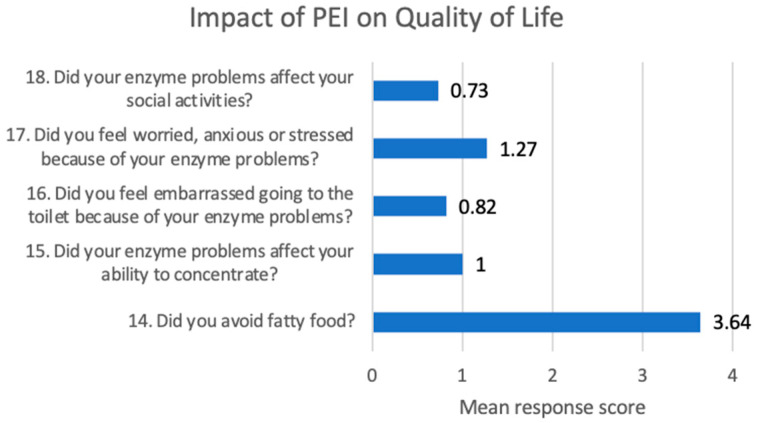
Impact of PEI on quality of life—at 2-month follow-up.

Upon analyzing the impact on quality of life among patients with a PEI-Q score ≥ 0.6, once again, the most affected domain was avoidance of fatty food consumption, with a mean score of 3.64, followed by worry, anxiety, or stress related to enzyme-related digestive problems, with a mean score of 1.27 (Figure 6).

No significant differences were noted between mean PEI-Q scores in patients with different severities of PEI according to FE-1 values (Table 3).

Regarding the symptoms related to PEI, those with PEI defined by an FE-1 value <200 µg/g more frequently reported symptoms such as abdominal pain, bloating, and flatulence. Those with FE-1 values <100 µg/g had higher scores on questions related to abdominal pain, bloating, foul-smelling gas, foul-smelling stools, and increased stool frequency.

### 3.3. Second Follow-Up Assessment (6 Months Post AP)

Out of the total number of patients included in the study, 19 patients returned for the second follow-up conducted at 6 months after the initial episode of acute pancreatitis (Table 4).

At the 6-month follow-up, the majority of patients (77%) had normal FE-1, while 23% had values between 100 and 199 µg/g. None of the patients showed severe exocrine pancreatic insufficiency, defined as an FE-1 value below 100 µg/g.

On the PEI-Q questionnaire, most respondents had scores below 0.6, indicating the absence of PEI; 4 patients (21%) had scores consistent with mild PEI (between 0.6 and 1.39); and 1 patient had a score equal to or greater than 1.8, suggestive of severe PEI.

**Figure 7:**
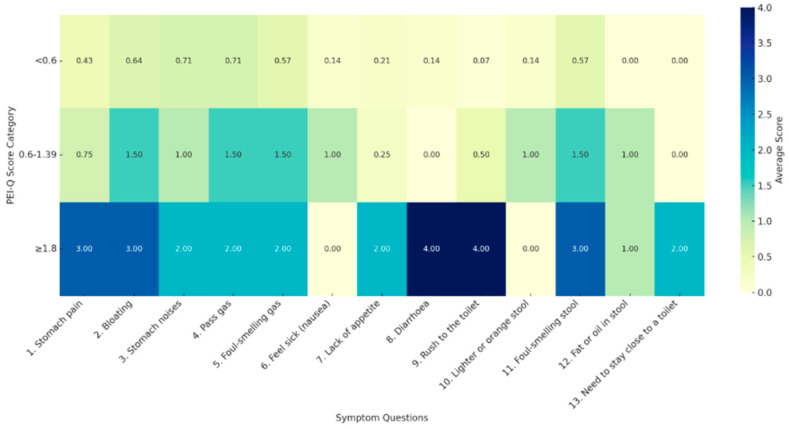
Heatmap of mean responses to PEI-Q questionnaire at 6 months follow-up.

Further, we analyzed the mean responses to each item in the PEI-Q questionnaire by grouping them according to PEI-Q score severity categories. There was a clear trend of increasing symptom severity in patients with severe PEI. (Figure 7).

**Figure 8:**
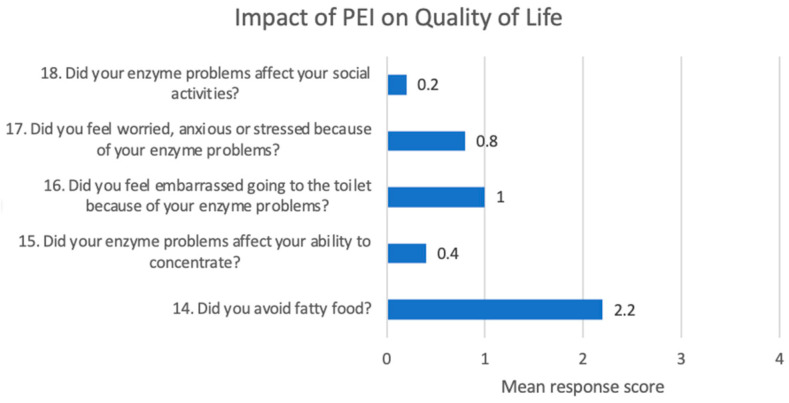
Impact of PEI on quality of life—at 6 months follow-up.

Regarding quality of life in patients with a PEI-Q score ≥ 0.6 at the 6-month follow-up, the item related to avoiding fatty foods had the highest mean response score. Social embarrassment and disease-related worry/stress showed moderate scores, followed by questions concerning impaired concentration and limitations in social activities (Figure 8).

Regarding PEI-Q score values by FE-1 category, no significant differences were observed between patient groups with different FE-1 levels (Table 5).

The most prominent symptoms among patients with FE-1 values < 200 µg/g at the 6-month evaluation were foul-smelling intestinal gas, flatulence, intestinal noises, nausea, and loss of appetite.

In order to explore potential differences, Table 6 summarizes the baseline characteristics of patients who completed the 6-month follow-up versus those lost to follow-up.

**Figure 9:**
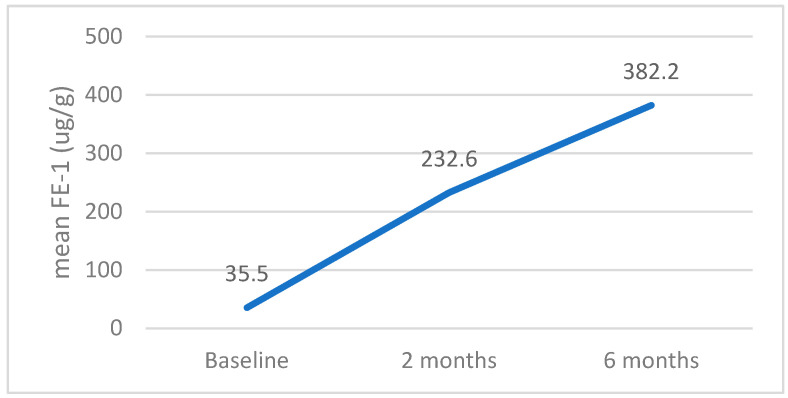
Dynamics of FE-1 values in patients with low values (<200 ug/g) at baseline.

Tracking the evolution of FE-1 values in patients with PEI at baseline (FE-1 < 200 ug/g), the trajectory was ascendent, with the increase recorded being statistically significant (*p* < 0.001)—Figure 9.

**Figure 10:**
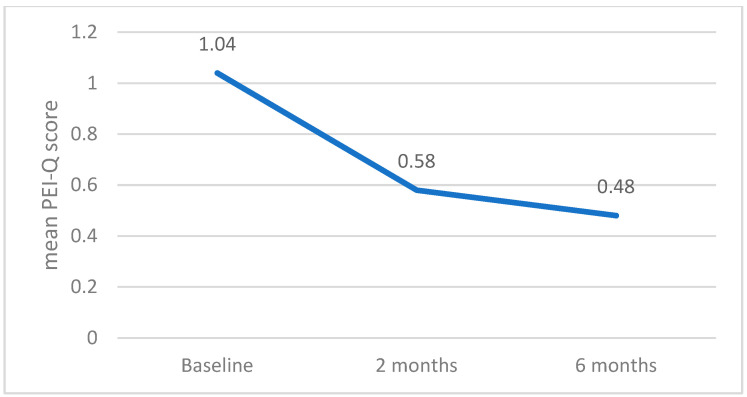
Dynamics of mean PEI-Q score from presentation to 2 and 6 months follow-up.

Similarly, the PEI-Q score showed a general downward trend from baseline (mean score 1.04 ± 0.61) to the 2-month (0.58 ± 0.52) and 6-month follow-up visits (mean 0.48 ± 0.46). The decrease of PEI-Q score was statistically significant (*p* < 0.001)—Figure 10.

Cronbach’s α values indicated high internal consistency of the PEI-Q questionnaire across all timepoints: 0.774 (0.676–0.852) at baseline, 0.880 (0.810–0.932) at 2 months, and 0.850 (0.738–0.928) at 6 months.

## 4. Discussion

Our study focuses on the prevalence of endocrine and exocrine insufficiency after AP flare, assessed by routine laboratory parameters and a validated patient-reported questionnaire, in correlation with features of the AP injury. Although patients were informed about the medical visits calendar and agreed to participate, a high rate of loss to follow-up was seen, with only 31.67% presenting to the 6-month follow-up. This may be due to several reasons: study burden (repeated questionnaires, blood and stool testing), logistical issues (patients living far from the center where they were treated for the AP flare and undergoing their follow-up in another facility), resolution of symptoms with perceived recovery (especially as most cases were mild pancreatitis), flare-up of comorbidities (taking precedence over AP follow-up), other complications, or even death. This low adherence to follow-up contributes to underdiagnosis of endo- and exocrine insufficiency post-AP and related long-term morbidity, and sets the need for reconsidering AP not just as an isolated medical episode, but one that requires follow-up for pancreatic dysfunction.

Regarding the etiology of AP, in our study, the most frequently encountered was biliary etiology (35%), followed by alcohol-related AP (18.3%), similar to other reports [18]. Although AP is uncommon as the first presentation of autoimmune pancreatitis [19,20,21], our cohort included one case of IgG4-related AP. This highlights the importance of considering rare etiologies of AP, as they may present particular profiles of PEI and endocrine insufficiency.

The majority of included patients had a mild form of AP (66.7%). An elevated leukocyte count was observed in 25% of patients, a parameter recognized as a predictor of disease severity, alongside CRP levels [22,23], both of which tend to rise in parallel with the increasing severity of AP [24]. At baseline, the mean CRP level was 159.57 mg/L. Although CRP levels progressively declined over time, they remained elevated at the 2-month follow-up (mean 26.88 mg/L). Close to normal values of CRP were observed at the second follow-up, 6 months after the AP flare.

Given that serum albumin has been identified as an independent risk factor for both disease severity and mortality in AP [25,26], we assessed its levels during the first hospitalization of our patients. The mean baseline serum albumin concentration was 3.65 ± 0.56 g/dl. Because serum albumin is significantly lower in patients with PEI [27], we continued monitoring albumin levels during follow-up visits, observing a progressive increase in its mean values, in parallel with clinical improvement and pancreatic recovery (4.2 g/dL at 2 months and 4.39 g/dL at 6 months, respectively).

We highlight the elevated value of mean fasting glucose in our cohort (120.75 ± 49.89 mg/dL), with more than half of the included patients having on-admission hyperglycemia (53.3%). This is important as serum glucose has been proven to be associated with increasing AP severity and mortality [28], as well as prolonged hospitalization [29]. It can also play a role in the recovery process after an episode of AP [30]. In our study, the mean fasting glucose normalized by the first follow-up visit (98.46 mg/dL), and continued to decline at the 6-month follow-up (96.55 mg/dL). In contrast, a systematic review and meta-analysis reported that 15% of patients developed DM within one year following an initial episode of AP, with this proportion rising to 40% at five years [8,31]. Hyperglycemia observed during the acute phase of AP is considered an adaptive response to metabolic stress induced by inflammation, in order to protect vital organs [29]. The predominance of mild AP forms in our study, alongside the longer follow-up duration and the larger sample size included in the referenced meta-analysis, may account for the discrepancies between findings.

FE-1 is the most widely used tool for diagnosing PEI. Although it is not considered the gold standard, it remains a practical and patient-friendly option due to its non-invasive nature, ease of use, time efficiency, and the fact that it is not influenced by ongoing pancreatic enzyme replacement therapy [32].

PEI as a complication of chronic pancreatic diseases has been investigated extensively and is primarily associated with chronic pancreatitis and cystic fibrosis [33]. The relation between AP and PEI is more and more recognized in the medical literature [34]. Although there are studies that found no correlation between the presence of PEI and the time elapsed since the acute episode of pancreatitis [35], other studies observed that PEI could be diagnosed in more than half of patients during hospitalization for AP (62%) [10], with a prevalence that tends to decline over time. Approximately one-third of patients continue to exhibit PEI in the longer term following the AP episode [33]. The difficulty of diagnosing PEI also comes from the fact that a decrease in pancreatic exocrine secretion is not always accompanied by a reduction in digestive capacity, due to pancreatic reserve and extra-pancreatic lipolysis [36].

Besides using FE-1 to guide clinical decisions, the novelty of our research lies in using a patient-reported questionnaire—PEI-Q, which has been previously validated in patients with chronic pancreatitis [13,15,37]. While currently available guidelines do not incorporate patient-reported outcomes (PRO) in the management of AP [38], and there are only a few validated questionnaires for pancreatitis [39], we consider that using simple direct questionnaires to assess patient symptoms might bring added value to FE-1 testing. The reduced sensitivity of FE-1 in mild to moderate PEI may lead to false-negative outcomes, contributing to delayed diagnosis and therapeutic intervention [11,40]. In our study, while FE-1 identified 23% of patients as having PEI at admission, when using the PEI-Q questionnaire, 75% of the patients had score results in favor of PEI. Of them, 25% had moderate and severe PEI according to PEI-Q score.

When analyzing the PEI-Q scores and FE-1 values, a negative correlation was observed with higher PEI-Q scores being seen in patients with lower FE-1 levels. At the first follow-up visit, the proportion of those with PEI confirmed by FE-1 level decreased to 15.2%, and the rate of PEI according to PEI-Q score decreased to 33.3%. However, we noted a significant increase in symptom severity at the 2-month follow-up, especially in patients with severe PEI according to PEI-Q score. Their main symptoms were diarrhea and fecal urgency, followed by abdominal pain, bloating, and modifications in stool color. At the second follow-up, the PEI appreciation by FE-1 level was 23%, while the proportion of PEI diagnosed by PEI-Q score was 26.3%. While a progressive decrease in PEI frequency according to PEI-Q scores was seen during follow-up (from 75% on-admission, to 33.3% and 26.3% at 2 and 6 months, respectively), this trend was not also seen with FE-1; this lack of consistency might be explained by several factors. First, the reduced adherence to follow-up was responsible for the low patient number at the 6-month assessment, potentially limiting the statistical power to detect trends in FE-1 values. Second, we observed that patients with low FE-1 during the initial hospitalization demonstrated a progressive improvement in pancreatic exocrine function during subsequent visits. We hypothesize that these patients may be more motivated to adopt and maintain a healthier lifestyle compared to those with preserved exocrine function, especially with regard to the active avoidance of risk factors, such as alcohol consumption and smoking. This group may also benefit from increased medical attention, with greater involvement of healthcare providers in their follow-up and management. Alternatively, the inconsistency may be the reflection of the presence of subclinical or undiagnosed features of CP in some patients, particularly those with ongoing exposure to harmful behaviors. Finally, delayed complications of AP, including progressive and late-onset exocrine dysfunction, cannot be ruled out as contributing factors.

Overall, in our study, there was a trend of decreasing PEI prevalence over time after an episode of AP, suggesting the pancreatic recovery potential after an acute injury. However, it is worth noting that PEI will persist in around one quarter of patients at follow-up and that the severity of symptoms augments over time in patients with severe exocrine insufficiency. In this context, some authors recommend annually checking for exocrine and endocrine insufficiency after AP [41].

A section of the PEI-Q questionnaire is dedicated to the impact of PEI on quality of life. The most affected domain was avoidance of fatty food consumption at all three moments of evaluation (baseline, 2 months, 6 months). This reflects a significant adaptive modification of patients’ lifestyle determined by the presence of PEI, with an important impact on their quality of life. Patients also describe anxiety, worry, and stress related to PEI symptoms, together with embarrassment, both impacting their general well-being. For this reason, an early diagnosis of PEI is encouraged in order to ensure early management to reduce the burden and to improve quality of life [16].

### Limitations

This study has several limitations to be acknowledged. First, the relatively small sample size may have limited the statistical power to detect certain associations and reduced the possibility of conducting detailed subgroup analyses. Future validation through larger, multicenter studies is therefore warranted.

Missing follow-up data is a recognized limitation in prospective observational studies. However, the use of multiple imputation allowed us to address this limitation and minimize potential bias. Our sensitivity analysis indicated no significant differences in key outcome measures between participants who completed follow-up and those with imputed data, supporting the validity of our imputation model. This strengthens the reliability of our findings regarding the evolution of exocrine and endocrine pancreatic function after AP.

Given the limited availability of baseline FE-1 measurements, these results should be interpreted as exploratory and hypothesis-generating.

A known limitation of PEI-Q score is that it was validated for assessing exocrine insufficiency in chronic pancreatitis, and its utility in AP remains insufficiently studied. Nevertheless, its application has lately expanded to other populations at risk of exocrine insufficiency, including patients with pancreatic surgery or DM [14,42].

Furthermore, while the present study focused on a 6-month follow-up period, we acknowledge that longer-term follow-up (e.g., 12 months or beyond) may provide further insights into the progression of pancreatic dysfunction after AP. Future research should aim to include extended follow-up and stratified analyses to better understand long-term outcomes and risk factors.

## 5. Conclusions

Acute pancreatitis should not be regarded as an isolated event. Long-term follow-up is essential, as acute pancreatic injury may lead to both endocrine and exocrine insufficiencies over time, each contributing to a decline in patients’ quality of life. Although with high prevalence at the onset of AP flare, PEI was noted in around one-fourth of patients according to PEI-Q score and FE-1 values at the 6-month follow-up. With regard to disturbances in glucose metabolism, hyperglycemia was seen in over half of patients during index admission, while at the 6-month follow-up, most patients were normoglycemic.

## Figures and Tables

**Table 1:** Patients’ baseline characteristics.

*Demographics*	*Value*
**Gender (n, %) (Male)**	29 (48.3%)
**Age, years (Mean** ± **SD)**	57.18 ± 13.3
***Risk factors:***	
**Smoking (n, %)**	15 (25%)
**Alcohol (n, %)**	21 (35%)
***Etiology of AP (n, %)***	
**Alcoholic**	11 (18.3%)
**Biliary**	21 (35%)
**Hypertriglyceridemia**	8 (13.3%)
**Idiopathic**	10 (16.7%)
**Post-ERCP**	9 (15%)
**Other**	1 (1.7%)
***Severity of AP (n, %)***	
**Mild**	40 (66.7%)
**Moderately-Severe**	15 (25%)
**Severe**	5 (8.3%)
***Previous DM (n, %)***	11 (18.3%)
***Biological parameters (Mean*** * ± **SD)/(n, %)***	
**Leukocyte count (×10^3^/mm^3^)**	11.85 ± 5.18
***Elevated leukocytes***	15(25%)
**Peak CRP (mg/L)**	159.57 ± 143.18
**Serum albumin (g/dL)**	3.65 ± 0.56
**Fasting Glucose (mg/dL)**	120.75 ± 49.89
***Elevated fasting glucose***	32 (53.3%)
**HbA1c (N = 36)**	6.23 ± 1.51
**HbA1c—Prediabetes/Diabetes**	10/10
***FE-1 (ug/g) (n,%)***	
**<100**	3 (11.5%)
**100** **–** **199**	3 (11.5%)
**≥200**	20 (77%)
***Imaging characteristics (Mean*** ± ***SD)/(n, %)***	
Balthazar score (N = 51)	A—3 (5.9%), B—3 (5.9%), C—20 (39.2%) D—4 (7.8%), E—21 (41.2%)
CTSI score (N = 51)	3.00 ± 1.74
***PEI-Q score (n, %*** ***)***	
**<0.6**	15 (25%)
**0.6** **–** **1.39**	30 (50%)
**1.4** **–** **1.79**	9 (15%)
**≥1.8**	6 (10%)

**Table 2:** Patients’ characteristics at first follow-up (2 months).

*Parameter*	*Value (Mean ± SD)*
**Fasting glucose (mg/dL)**	98.46 ± 33.94
***Elevated fasting glucose (n, %)***	11 (33.3%)
**HbA1c** ** (%)**	5.23 ± 0.44
**CRP (mg/dL)**	12.09 ± 36.66
***Elevated CRP (n, %)***	9 (27.3%)
**Albumin (g/dL)**	4.2 ± 0.41
**FE-1 (ug/g)**	
***FE-1—category* (n, %)**	
**<100**	3 (9.1%)
**100–199**	2 (6.1%)
**≥200**	28 (84.8%)
**PEI-Q score (n, %)**	
**<0.6**	22 (66.7%)
**0.6–1.39**	9 (27.3%)
**1.4–1.79**	1 (3%)
**≥1.8**	1 (3%)

**Table 3:** Mean PEI-Q score in different subgroups of FE-1 values—2-month follow-up.

FE-1 (ug/g)	Mean PEI-Q Score
<100	0.89
100–199	0.22
≥200	0.59

**Table 4:** Patients’ characteristics at second follow-up visit (6 months).

*Parameter*	*Value (Mean ± SD)*
**Fasting glucose (mg/dL)**	96.55 ± 11.73
**HbA1c** ** (%)**	5.83 ± 0.72
**CRP** ** (mg/dL)**	3.68 ± 3.29
**Albumin (g/dL)**	4.39 ± 0.33
**FE-1** ** (ug/g)**	
**<100**	0 (0%)
**100–199**	3 (23%)
**≥200**	10 (77%)
**PEI-Q score (n,%)**	
**<0.6**	14 (73.7%)
**0.6–1.39**	4 (21.0%)
**1.4–1.79**	0 (0.0%)
**≥1.8**	1 (5.3%)

**Table 5:** Mean PEI-Q score in different subgroups of FE-1 values—6 months follow-up.

FE-1 (ug/g)	Mean PEI-Q Score
<100	0
100–199	0.56
≥200	0.5

**Table 6:** Baseline characteristics of patients who completed the 6-month follow-up (completers) versus those lost to follow-up (drop-outs).

	Completers	Drop-Outs
**Total**	19	41
**Age, years (mean ± SD)**	57.31 ± 11.39	57.12 ± 14.34
**Males (n, %)**	12 (63.16%)	17 (41.45%)
**Risk factors**		
***Tabac (n, %)***	7 (36.84%)	8 (19.51%)
***Alcohol (n, %)***	9 (47.37%)	12 (29.27%)
**Previous DM (n, %)**	2 (10.52%)	9 (21.95%)
**Etiology (n, %)**		
***Alcohol***	7 (36.84%)	4 (9.76%)
***Biliary***	5 (26.32%)	16 (39.02%)
***Hypertriglyceridemia***	2 (10.52%)	6 (14.63)
***Idiopatic***	5 (26.32%)	5 (12.20%)
***Other***	0	1 (2.44%)
***Post-ERCP***	0	9 (21.95%)
**Severity of AP (n, %)**		
***Mild***	9 (47.37%)	31 (75.61%)
***Moderately severe***	8 (42.11)	7 (17.07%)
***Severe***	2 (10.52%)	3 (7.32%)

## Data Availability

Datasets are available from the corresponding author.

## Supplementary Materials

The following supporting information can be downloaded at: https://www.mdpi.com/article/10.3390/jcm14144932/s1.
