# Supplementary material for: From Inflammation to Dysfunction: The Impact of a First Acute Pancreatitis Episode on Pancreatic Function
Source: J Clin Med. 2025 Jul 11;14(14):4932. doi: 10.3390/jcm14144932 (PMC12295321; doi:10.3390/jcm14144932)
Supplement: Supplementary file 1 [file jcm-14-04932-s001.zip › jcm-3691458-supplementary.pdf]

PEIQ

PANCREATIC EXOCRINE INSUFFICIENCY QUESTIONNAIRE

(ENGLISH)

January 2019

Abbott

PEI-Q
PANCREATIC
EXOCRINE INSUFFICIENCY
QUESTIONNAIRE

PEI-Q scoring sheet *[for administration use only]*

- Each item is assigned a score from 0-4 based on the response selected by the patient.
- Each domain and total score is calculated by taking an average (mean), as shown in the table below.
- The ***total symptom score (mean)*** can be calculated for all respondents to provide information to help doctors to determine a diagnosis of PEI, while the ***total summary score (mean)*** should only be calculated for respondents who have been diagnosed with PEI.
- Domain scores can only be calculated if more than 50% of items are completed within each domain (i.e. four or more items in the abdominal symptoms domain, three or more items in the bowel movement symptoms domain, and three or more items in the impacts domain).
- Total scores can only be calculated if two or more domain scores are present for each patient.
- If more items/domains are missing, the domain/total score cannot be calculated and will be considered missing.
- Evidence suggests that patients with a ***total symptom score (mean)*** of greater than or equal to **0.60** is consistent with a diagnosis of PEI, if the individual does not have a diagnosis of another gastrointestinal condition such as irritable bowel syndrome with diarrhoea.
- Evidence also suggests that a ***total symptom score (mean)*** of greater than or equal to **1.8** is indicative of severe or poorly controlled PEI, scores of **1.4-1.8** are indicative of **moderate** PEI symptoms and scores of **0.60-1.4** are indicative of minimal or **mild** PEI symptoms.

Scoring

DOMAIN (MEAN)	FORMULA	PLEASE WRITE THE PATIENT'S SCORE HERE
Abdominal symptoms (A)	A= Sum of scores of items 1 to 7 / 7	A =
Bowel movement symptoms (B)	B= Sum of scores of items 8 to 13 / 6	B =
Total symptom score	*(Abdominal symptoms domain score (A) + bowel movement symptoms domain score (B))* / 2	(A+B)/2 =
Impacts (C)	C= Sum of scores of items 14 to 18 / 5	C =
**Total summary score (PEI patients only)**	*(Abdominal symptoms domain score (A) + bowel movement symptoms domain score (B) + impacts domain score (C))* / 3	(A+B+C)/3 =

To protect the integrity of the instrument, the PEI-Q including the user manual, scoring algorithm and any versions or translations of the above, and all associated intellectual property rights, are owned by Abbott.

January 2019

Abbott

PEI-Q

PANCREATIC EXOCRINE INSUFFICIENCY QUESTIONNAIRE

This questionnaire asks about problems you may experience if you do not produce enough enzymes to digest your food.

Note to administrator: It is recommended that the Bristol Stool Form Scale is administered alongside the PEI-Q.

NAME: ______________________

DATE: ☐☐ ☐☐ 20☐☐
DAY MONTH YEAR

ABDOMINAL SYMPTOMS

		NO NOT AT ALL	YES, A LITTLE BIT	YES, SOME	YES, QUITE A BIT	YES, A LOT	SCORE
1.	In the past 7 days, did you have **stomach pain?**	☐ 0	☐ 1	☐ 2	☐ 3	☐ 4	☐
2.	In the past 7 days, did you **feel** bloated (your stomach feeling **tight** and **full**)?	☐ 0	☐ 1	☐ 2	☐ 3	☐ 4	☐
3.	In the past 7 days, did your **stomach make noises?**	☐ 0	☐ 1	☐ 2	☐ 3	☐ 4	☐
4.	In the past 7 days, did you **pass gas**?	☐ 0	☐ 1	☐ 2	☐ 3	☐ 4	☐
5.	In the past 7 days, when you **passed gas** did it **smell** very **bad**?	☐ 0	☐ 1	☐ 2	☐ 3	☐ 4	☐
6.	In the past 7 days, did you **feel sick** (but didn't actually vomit/throw up)?	☐ 0	☐ 1	☐ 2	☐ 3	☐ 4	☐
7.	In the past 7 days, did you have a **lack of appetite?**	☐ 0	☐ 1	☐ 2	☐ 3	☐ 4	☐
						**Sum of abdominal symptom scores:**	☐
						**Mean abdominal symptom domain score (A):**	☐

BOWEL MOVEMENT SYMPTOMS

		NO NOT AT ALL	YES, A LITTLE BIT	YES, SOME	YES, QUITE A BIT	YES, A LOT	SCORE
8.	In the past 7 days, did you have **diarrhoea (watery poo)**?	☐ 0	☐ 1	☐ 2	☐ 3	☐ 4	☐
9.	In the past 7 days, did you feel the need to **rush to the toilet** to have a **bowel movement (have a poo)**?	☐ 0	☐ 1	☐ 2	☐ 3	☐ 4	☐
10.	In the past 7 days, did your **poo** look **lighter** or **orange** in colour?	☐ 0	☐ 1	☐ 2	☐ 3	☐ 4	☐
11.	In the past 7 days, when you had a **poo** did it **smell** very **bad**?	☐ 0	☐ 1	☐ 2	☐ 3	☐ 4	☐
12.	In the past 7 days, did you see or have **fat or oil** in your **poo** or on the **toilet paper**?	☐ 0	☐ 1	☐ 2	☐ 3	☐ 4	☐

		NO NOT AT ALL	YES, A LITTLE BIT	YES, MODERATELY	YES, QUITE A BIT	YES, EXTREMELY	SCORE
13.	In the past 7 days, did you feel you needed to be **close to a toilet** because of your **enzyme problems**?	☐ 0	☐ 1	☐ 2	☐ 3	☐ 4	☐
						**Sum of bowel movement symptom scores:**	☐
						**Mean bowel movement symptom score (B):**	☐
						**MEAN TOTAL SYMPTOM SCORE ([A+B]/2):**	☐

To protect the integrity of the instrument, the PEI-Q including the user manual, scoring algorithm and any versions or translations of the above, and all associated intellectual property rights, are owned by Abbott.

January 2019

Abbott

PEIQ
PANCREATIC
EXOCRINE INSUFFICIENCY
QUESTIONNAIRE

Only complete if you have been diagnosed with Pancreatic Exocrine Insufficiency (PEI)

IMPACTS	NO NOT AT ALL	YES, A LITTLE OF THE TIME	YES, SOMETIMES	YES, MOST OF THE TIME	YES, ALL OF THE TIME	SCORE
14. In the past 7 days, did you avoid **fatty food**?	☐ 0	☐ 1	☐ 2	☐ 3	☐ 4	☐
15. In the past 7 days, did your **enzyme problems** affect your ability to **concentrate**?	☐ 0	☐ 1	☐ 2	☐ 3	☐ 4	☐

	NO NOT AT ALL	YES, A LITTLE BIT	YES, MODERATELY	YES, QUITE A BIT	YES, EXTREMELY	SCORE
16. In the past 7 days, did you feel **embarrassed going to the toilet** because of your **enzyme problems**?	☐ 0	☐ 1	☐ 2	☐ 3	☐ 4	☐
17. In the past 7 days, did you feel **worried, anxious** or **stressed** because of your **enzyme problems**?	☐ 0	☐ 1	☐ 2	☐ 3	☐ 4	☐

	NO NOT AT ALL	YES, A LITTLE OF THE TIME	YES, SOMETIMES	YES, MOST OF THE TIME	YES, ALL OF THE TIME	SCORE
18. In the past 7 days, did your **enzyme problems** affect your **social activities**?	☐ 0	☐ 1	☐ 2	☐ 3	☐ 4	☐
**Sum of impact scores:**						☐
**Mean impact domain score (C):**						☐
**MEAN TOTAL SUMMARY SCORE (PEI PATIENTS) ([A+B+C]/3):**						☐

To protect the integrity of the instrument, the PEI-Q including the user manual, scoring algorithm and any versions or translations of the above, and all associated intellectual property rights, are owned by Abbott.

January 2019

Abbott
